# Climate Change Alters Elevational Distribution Patterns of *Cormus domestica* Habitat

**DOI:** 10.1002/ece3.73602

**Published:** 2026-04-29

**Authors:** Qianjiang Li, Zhuoling Li, Haozheng Li, Bohao He, Lorenzo Mari

**Affiliations:** ^1^ School of Architecture Urban Planning Construction Engineering Polytechnic University of Milan Piacenza Italy; ^2^ Forestry and Parks Bureau of Handan Handan China; ^3^ Department of Electronics, Information and Bioengineering Polytechnic University of Milan Milan Italy

**Keywords:** climate change, elevational shifts, habitat distribution, species migration

## Abstract

The biodiversity of temperate forests is severely threatened by climate change, with many species facing loss of suitable habitats or local extinction. Species that are highly sensitive to climate change, especially those with low dispersal ability, pose significant challenges. A comprehensive overview of the impact of climate change on the elevational shifts of temperate trees is still missing. In this study, we used species distribution models to study range shifts of *Cormus domestica* (L.) in Italy under current and future scenarios (SSP2‐4.5 and SSP5‐8.5), specifically focusing on the species' elevational shifts under future climate change. Our models project a considerable loss of habitat suitability over time (32%–68% by 2100). Annual mean temperature is the primary limiting variable, with the margin of the optimal temperature range at about 12°C. We found that climate change will alter the spatial configuration of suitable areas, with newly gained habitats always at higher elevations than lost ones. This result implies a trend of suitable areas shifting toward higher elevations, but in a way that cannot compensate for habitat loss. Our results point to the urgent need to strengthen conservation efforts aimed at improving the climate resilience of low‐elevation species.

## Graphical abstract

This study utilizes species distribution models to project the habitat shifts of *Cormus domestica* in Italy, predicting a substantial range reduction of 32%–68% by 2100 driven primarily by rising annual mean temperatures. The results indicate a significant shift of suitable habitats toward higher elevations, creating a scenario where the necessary migration speed exceeds the species' natural dispersal ability. These findings suggest a high extinction debt and highlight the urgent need for conservation strategies focused on vulnerable low‐elevation populations.

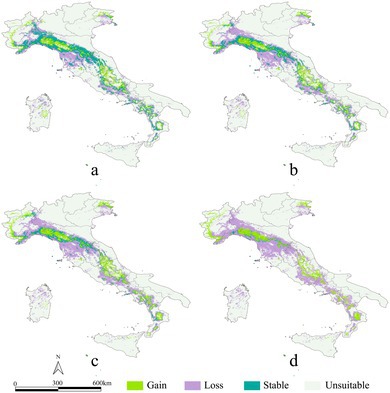

## 1 Introduction

Global climate change significantly affects the suitable habitats of species and biological communities (Bellard et al. 2012; Parmesan and Yohe 2003). Human activities have unequivocally intensified global warming through greenhouse gas (GHG) emissions, causing global surface temperatures between 2011 and 2020 to rise by 1.1°C compared to pre‐industrial (1850–1900) levels (Lee et al. 2023). Between 1970 and 2010, CO_2_ emissions from fossil fuel use and industrial activities accounted for approximately 78% of all GHG emissions (Pachauri et al. 2014). Climate change has also triggered extreme weather events, including droughts and floods, directly harming biodiversity and ecosystems (Lee et al. 2023). Rising global temperatures increase the risk of species extinction, with an estimated 7.9% of species potentially facing extinction because of climate change (Urban 2015; Wiens and Zelinka 2024). Under current GHG emission trends, approximately one‐sixth of global species are projected to be threatened (Urban 2015). Warming temperatures have led to shifts in species ranges, as climate change may alter environmental conditions to an extent that exceeds a species' physiological tolerance limits, thereby reducing the extent of suitable habitats. These changes in the biotope induce habitat range shifts that can, in turn, trigger adaptive mechanisms in the fundamental niche of a species (Colwell and Rangel 2009) and lead to a reshuffling of a species' position within its climatic niche (Antão et al. 2022).

Trees are under immense stress from climate change. Persistent global climate change, including rising CO_2_ levels and reduced precipitation, profoundly impacts plant photosynthesis, phenology, nutrient composition, and phenotypes (Chaudhry and Sidhu 2022). *Cormus domestica* (L.) Spach (typically classified as *Sorbus domestica* (L.) in the past), also known as the common service tree, is a fruit‐bearing tree in the Rosaceae family, a temperate forest species pollinated by insects (Kamm et al. 2009), with potential as an urban and landscape ornamental plant (Schmucker et al. 2024). This species can adapt to dry, mild climates, which underscores its relevance in the context of future climate change adaptation in some regions, such as Northern Europe. As a forest fruit tree, its fruits serve as a food source (nowadays mostly for animals) and are also valued for their antioxidant properties (Termentzi et al. 2009). Additionally, its wood can be used to produce high‐quality hardwood veneer, serving the timber industry (Piagnani et al. 2018). However, there is still a lack of research on the effects of climate change on the habitat of this species. Habitat loss due to climate change represents a major threat to *C. domestica* (Afifi et al. 2023). Changes in global temperature gradients, altered precipitation patterns, and extreme weather events pose significant threats to its survival. *C. domestica* may respond to these threats through migration, facilitated by its reproductive strategies. This species relies on sexual reproduction and seed dispersal to colonize new suitable habitats, thereby altering its habitat range (Rasmussen and Kollmann 2004). *C. domestica* exhibits high genetic diversity, promoting adaptation to new habitats through natural selection and increasing the likelihood of successful dispersal (George et al. 2015). As a mountainous forest species, *C. domestica* is likely to shift to higher elevation under future climate scenarios, tracking more favorable conditions in terms of temperature and/or precipitation levels. Through its dispersal capacity, *C. domestica* may establish new habitats in these higher‐elevation regions.

Species migrate to new habitats in response to environmental changes. Studies have shown that 84% of species' habitat shifts align with climate change, with migrations typically moving toward higher latitudes and/or elevations (Hickling et al. 2006; C. D. Thomas 2010). Research indicates that the median migration distance of species is 11 m per decade toward higher elevations and 16.9 km per decade toward higher latitudes (Chen et al. 2011). To adapt to the climate changes of the 21st century, however, plant species would need to latitudinally migrate at rates of 30–50 km per century. Therefore, the median migration distance falls well short of this requirement (Chen et al. 2011). Historical and current data have been used to analyze plant migration distances to higher elevations. For instance, a study of 171 forest tree species in Western Europe during the 20th century found that the mean elevation at which their habitat suitability peaked increased by an average of 29 m per decade (Lenoir et al. 2008). Similarly, research on vascular plants in the Alps revealed a median migration distance of 12.9 m per decade for 52 species (Lenoir et al. 2008). Trees often face challenges in migration. Habitat fragmentation and limited dispersal capacity can hinder their migration speed, resulting in habitat contraction or even local extinction. Exploring species' tendencies to migrate to higher elevations under future climate change is critical for conservation strategies that enable species to track shifting climatic requirements.

Species distribution models (SDMs) are widely employed to project the impacts of climate change on species habitat shifts and to interpret the contributions of environmental factors in eliciting such response (Elith and Leathwick 2009; He et al. 2022, 2023). In studies on forest tree species, SDMs have been used to project future habitat distributions under climate change, often revealing reductions in suitable habitats (Iverson and Prasad 2002). For example, research on larch has used SDMs not only to project habitat changes but also to provide insights into the relative importance of environmental factors (Leng et al. 2008). Expanding on these applications, studies on tulip trees have combined SDM projections with analyses of physiological indicators related to stress tolerance (Shen et al. 2022). Although these studies have offered valuable insights into the habitat distribution of the target species, they have not thoroughly explored elevational shifts driven by climate change. To fill this gap, in the present study, we apply SDMs to project the future habitat distribution of the common service tree, *C. domestica*, in Italy, focusing specifically on possible elevational shifts under changing climate conditions. SDMs have become indispensable tools for projecting climate change effects on species habitats and play a crucial role in conservation planning and decision‐making (Guisan et al. 2013).

The impact of climate change on the habitat distribution of *C. domestica* remains uncertain, which results in conservation strategies for this species' habitat that fail to adequately incorporate temporal and spatial considerations. Therefore, our study aims to (1) analyze the current and future habitat distribution and changes of *C. domestica* under different climate scenarios; (2) identify the key environmental factors influencing its distribution; and (3) project potential future trends of suitable habitats shifting to higher elevations. Predicting the habitat changes of *C. domestica* is essential for mitigating habitat degradation and conserving biodiversity, thereby safeguarding its ecological value in the future.

## 2 Materials and Methods

### 2.1 Species Occurrence Data

The study area encompasses the entire territory of Italy, covering a total area of 302,109.57 km^2^. Italy is situated between latitudes 36°28′ N and 47°6′ N, and longitudes 6°38′ E and 18°31′ E. Records of *C. domestica* occurrences were obtained from Global Biodiversity Information Facility (GBIF; https://www.gbif.org/) and Portal to the Flora of Italy (https://dryades.units.it/floritaly/index.php). We compiled a dataset of observation occurrences (*n* = 211) from 1970 to 2024, removing duplicate records and those with coordinate uncertainty. We verified the locations of species occurrence records using latitude and longitude coordinates to prevent obvious positional errors, such as records that incorrectly appeared at sea or within lakes. Spatial autocorrelation among sampling points can introduce environmental bias, leading to overfitting and inflated estimates of model performance. We used the Spatially Rarefy Occurrence Data tool of SDMtoolbox Pro v0.9.1 in ArcGIS Pro 3.2 to reduce spatial autocorrelation by ensuring that each grid cell of raster data contained only one occurrence record, resulting in a final dataset of 136 records (Figure S1).

### 2.2 Environmental Data

We preliminarily considered eight environmental variables, including bioclimatic and landscape factors, to develop models assessing the habitat distribution of *C. domestica* (Table 1). To ensure that the selection of environmental variables aligns with the ecological adaptability of the species, we selected key climate and landscape factors on the basis of the habitat requirements of our target species (Paganová 2007).

**TABLE 1:** Description of the environmental variables considered in the study.

Variables	Description	Unit
Bio‐1	Annual mean temperature	°C
Bio‐8	Mean temperature of wettest quarter	°C
Bio‐12	Annual precipitation	mm
Bio‐14	Precipitation of the driest month	mm
SOC	Soil organic carbon	dg/kg
River	Distance from the closest river	km
LULC	Land use and land cover: cropland, forest, grassland, urban, barren, and water	/
Elevation	Elevation above sea level	m

Bioclimatic variables were sourced from the WorldClim v2.1 database (https://www.worldclim.org/). In addition to using the annual mean temperature (Bio‐1) and annual precipitation (Bio‐12) to examine the effects of temperature and precipitation on this species, we considered the precipitation of the driest month (Bio‐14) because drought poses a significant threat to seedling growth (Kunz et al. 2016). As the rainy season in Italy occurs primarily in autumn and winter, we used the mean temperature of the wettest quarter (Bio‐8) to explore the temperature conditions required for the growth of this species under sufficiently moist conditions, thereby accounting for the potential threat posed by low winter temperatures. These bioclimatic variables capture the species' temperature and moisture regimes relevant to physiological tolerance, which are critical factors in determining the adaptability and distribution of *C. domestica* .

The landscape variables included soil organic carbon (SOC), the distance from the closest river (River), land use and land cover (LULC, a categorical variable), and elevation. The selection of these landscape variables was based on their significant influence on habitat conditions. SOC is strongly associated with soil fertility, aeration, and organic matter content, all of which directly affect nutrient availability and the root environment for plants. The distance to rivers relates to soil moisture, which is essential for species survival. LULC influences habitat structure and community composition, significantly affecting habitat quality. Elevation was included to analyze possible variations in habitat elevation under climate change. Elevation was taken from WorldClim v2.1. Soil organic carbon (SOC) was derived from the SoilGrids database (https://soilgrids.org/), published by the International Soil Reference and Information Center (ISRIC). The distance to rivers was calculated on the basis of the Italian Hydrological Network database (Yan et al. 2022). LULC was derived from publicly available databases (Zhang et al. 2023).

To prevent overfitting, we performed pairwise spatial correlation analysis between the predictor variables and excluded elevation because of its strong anticorrelation (Pearson correlation coefficient = −0.912) with annual mean temperature (Bio‐1). The final set of predictors thus included seven variables, all of which were characterized by pairwise Pearson spatial correlation lower than 0.8 (Figure S2). As for future conditions, we selected the 2041–2060 (referred to as “2060”) and 2081–2100 (referred to as “2100”) time periods to capture both mid‐term conservation planning horizons (about one generation time for *C. domestica* ) and end‐of‐century trajectories relevant for long‐term persistence assessment. We adopted the Shared Socioeconomic Pathway (SSP) scenarios from the Intergovernmental Panel on Climate Change (IPCC) Sixth Assessment Report (AR6), focusing on an intermediate greenhouse gas (GHG) emissions scenario (SSP2‐4.5, “middle of the road”) and an extremely high GHG emissions scenario (SSP5‐8.5, “fossil‐fueled development”). SSP2‐4.5 represents moderate mitigation efforts, limiting global temperature rise to less than 3°C compared to pre‐industrial levels, whereas SSP5‐8.5 reflects high economic growth and heavy fossil fuel dependence, leading to an extreme temperature rise exceeding 4°C by the end of the 21st century.

The WorldClim dataset includes multiple climate data models and is widely used in species distribution studies. In addition, it has demonstrated better performance in downscaling comparisons with the CHELSA dataset in the Italian region (Fick and Hijmans 2017; Delle Monache et al. 2024). To minimize uncertainties associated with a single climate model, we utilized three global climate models (GCMs) from WorldClim: CMCC‐ESM2, GISS‐E2.1, and INM‐CM5. These models have been widely evaluated and used and have demonstrated reliable performance (Volodin et al. 2017; Kelley et al. 2020; Lovato et al. 2022). The future values of the climate variables were taken as the arithmetic means of the outputs of these models. This ensemble approach improves the reliability and robustness of climate projections, offering a more accurate assessment of the potential impacts of future climate change on species habitat distribution.

All data were standardized to the WGS 1984 coordinate system and processed at a spatial resolution of 30 s (approximately 1 km). National and regional administrative boundaries of Italy (Figure S4) were provided by the Italian National Institute of Statistics (https://www.istat.it/).

### 2.3 Species Distribution Model

In this study, we utilized the biomod2 v.4.2.6 package in R 4.3.2 to develop SDMs to predict the habitat distribution of *C. domestica*, our target species. According to Barbet‐Massin et al. (2012), two sets of pseudo‐absences were generated at a 1:1 ratio with the occurrence records (Figure S3). For single‐model development, we trained independent SDMs employing the following machine‐learning algorithms: Artificial Neural Networks (ANN), Classification Tree Analysis (CTA), Flexible Discriminant Analysis (FDA), Generalized Additive Models (GAM), Gradient Boosting Machine (GBM), Generalized Linear Model (GLM), Multivariate Adaptive Regression Splines (MARS), Maximum Entropy (MaxEnt), Random Forest (RF), Surface Range Envelope (SRE), and eXtreme Gradient Boosting (XGBoost). Model performance was evaluated using random 10‐fold cross‐validation, with 80% of the data allocated to the training set and 20% to the test set in each iteration. To construct an ensemble model, we selected a subset of these single model runs by identifying those in which the True Skill Statistic (TSS, a metric subsuming both sensitivity and specificity) value on the basis of the test set exceeded 0.7. We evaluated the performance of both single and ensemble models on the test set according to the area under the curve (AUC) of the receiver operating characteristic curve and the TSS. We calculated the contribution and response curves of environmental variables within the ensemble model. Each environmental variable was randomly permuted twice, and the mean decrease in model performance was calculated as the basis for ranking variable importance. Response curves for environmental variables were calculated by predicting habitat occurrence probabilities at evenly spaced values of each variable. We applied the threshold corresponding to the maximum TSS to convert the habitat suitability (i.e., probability of presence) maps into a binary classification of suitable and unsuitable habitats (Liu et al. 2005). The spatial extent of suitable habitat was then calculated to compare the changes projected across different climate scenarios.

### 2.4 Habitat Change and Elevation Shift Assessment

We used Italy's administrative divisions to quantify the area of suitable habitat within each region. To compare current and future habitat distributions, we classified pixels as “gained habitat” if currently unsuitable but suitable under future scenarios, and “lost habitat” if currently suitable but unsuitable under future scenarios. “Stable habitat” remained suitable across all time periods. The maximum, minimum, average, and median elevation values of current and future habitats were calculated to assess a possible shift in the elevational distribution of *C. domestica* . We also compared the elevations of gained and lost habitats under future climate scenarios to identify possible elevational shifts. To assess elevation differences between current and future suitable habitats, we performed a two‐way ANOVA (analysis of variance), followed by Tukey's honest significant difference (HSD) test to identify significant pairwise differences (Abdi and Williams 2010).

## 3 Results

### 3.1 Current and Future Habitat Distribution

**FIGURE 1:**
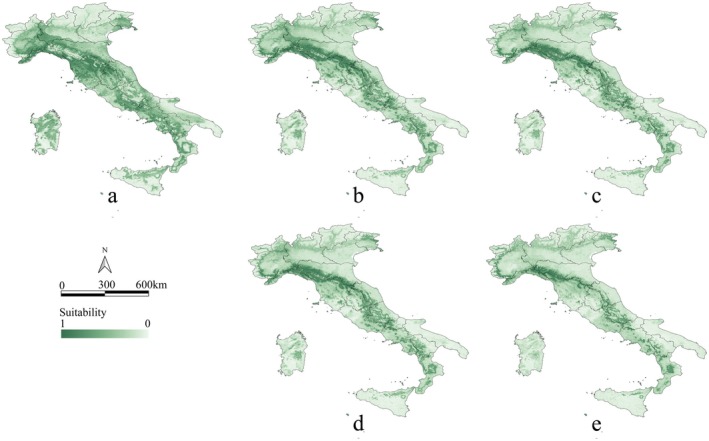
Habitat suitability maps for *C. domestica* . (a) Current conditions; (b) SSP2‐4.5 in 2060; (c) SSP5‐8.5 in 2060; (d) SSP2‐4.5 in 2100; and (e) SSP5‐8.5 in 2100. Suitability values range between 0 (minimum suitability) and 1 (maximum suitability).

**FIGURE 2:**
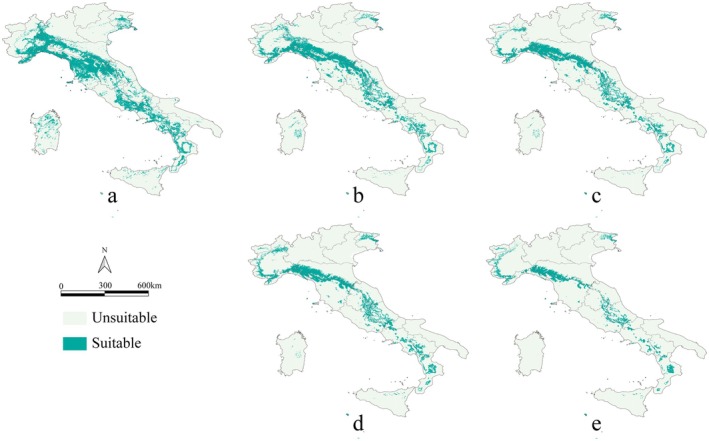
Suitable habitat maps for *C. domestica* . (a) Current conditions; (b) SSP2‐4.5 in 2060; (c) SSP5‐8.5 in 2060; (d) SSP2‐4.5 in 2100; and (e) SSP5‐8.5 in 2100. Suitable areas (on the basis of the maximum TSS) are represented in green, whereas gray indicates areas where environmental conditions are not suitable for *C. domestica* .

The ensemble model showed the highest performance (AUC = 0.91 ± 0.03, TSS = 0.75 ± 0.05) and could more accurately simulate the current spatial distribution of suitable habitat for *C. domestica* (Table S1). The standard deviations of TSS and AUC for all single models are much higher than the ensemble model's, which demonstrates the greater robustness of the latter. The habitat prediction results under current climate conditions (Figures 1a and 2a) indicate that suitable areas for *C. domestica* are primarily concentrated in the Apennines, a mountain range extending about 1200 km from northwest to southwest Italy. Toscana region (central Italy, see Figure S4) contains the largest extension of *C. domestica* habitats, covering 14,713 km^2^ (64% of the region) and accounting for approximately 22% of the total areas that are projected to be suitable for *C. domestica* in the whole country (Table S2). In northern Italy, Emilia‐Romagna and Piemonte regions have habitat areas comparable to each other, namely 6622 and 6526 km^2^ (29% and 26% of the region areas), each representing approximately 10% of the total habitat coverage at the country scale. In northern Italy, Liguria also strongly contributes to *C. domestica* habitats (4213 km^2^, 78% of the whole region), mainly along the coast, hosting about 6% of the total suitable area in the country. In central Italy, besides Toscana, suitable habitats are primarily located in Lazio (5044 km^2^, corresponding to 29% of the region and contributing to 7% of the suitable area at the country scale). In southern Italy, Campania and Calabria exhibit the most prominent habitat coverage, with suitable areas of 5441 and 4076 km^2^ (40% and 27% of the region's area), corresponding to 8% and 6% of total Italian habitats, respectively.

### 3.2 Future Changes in Suitable Habitat Distribution

We project significant reductions in suitable habitat areas for *C. domestica* in future climate scenarios (Figures 1b–e and 2b–e) compared with current conditions (Figures 1a and 2a). Under SSP5‐8.5 (Figure 2c,e), habitat loss is notably more severe than under SSP2‐4.5 (Figure 2b,d). Under SSP5‐8.5, the habitat area is projected to decrease in 2060 by 30,671 km^2^, around 45% of the current habitat area. In the same scenario, the loss in 2100 will reach 46,653 km^2^, representing 68% of the current habitat area (Table S3). Habitat loss is primarily concentrated in the Toscana region, with approximately 9068 km^2^ of habitat loss under SSP2‐4.5 in 2060. This figure is projected to become increasingly more severe under continued climate change (Table S4). Significant habitat loss is also observed in Emilia‐Romagna, Lombardia, Lazio, and Liguria, with even more pronounced losses under SSP5‐8.5 for 2060 and 2100. When comparing current conditions and SSP5‐8.5 in 2100, suitable habitats are expected to disappear almost entirely in regions such as Piemonte, Campania, Lazio, Lombardia, Friuli‐Venezia Giulia, Basilicata, Marche, Molise, Abruzzo, Puglia, Veneto, Umbria, Sardegna, and Sicilia. However, under future climate scenarios, new suitable areas are also projected to emerge. These newly established habitats are primarily located in the Apennine Mountains, within the regions of Tuscana and Emilia‐Romagna. Additionally, parts of the western Alps in the Piemonte region are anticipated to become suitable. In central and southern Italy, the new habitats are also predominantly distributed along the Apennine range.

### 3.3 Feature Contribution

The ranking of environmental variable contributions in influencing habitat prediction is shown in Figure 4a. The response curves (b–h) illustrate the relationship between each environmental variable and the habitat suitability for the target species. According to our projections, annual mean temperature (Bio‐1) is the most important variable determining the habitat suitability for *C. domestica* , followed by the mean temperature of the wettest quarter (Bio‐8) and the annual precipitation (Bio‐12). Habitat suitability exceeds 0.5 in a well‐defined range of annual mean temperature (11°C–15°C) and mean temperature of the wettest quarter (4°C–16°C), and for annual precipitation above a threshold of approximately 500 mm. As for the other variables, the response of the species' probability of occurrence to variations in soil organic content (SOC) is not particularly pronounced; however, a relatively higher suitability is observed within a SOC range of 500–850 dg/kg. For the precipitation of the driest month (Bio‐14), the species' probability of occurrence remains mostly above 0.5, reaching a peak at approximately 30 mm. The response curves associated with the distance from the closest river (River) and land use and land cover (LULC) show relatively little variation.

### 3.4 Elevational Shifts

We recorded the elevation data of suitable habitats for *C. domestica* under current conditions and two future climate scenarios at two different time points. The results reveal a clear shift of suitable habitats to higher elevations under future climate conditions (Figure 5a). Under current and projected future climate conditions, the mean elevations of suitable habitats move from 427 to 695–807 m in 2060, and 874–1075 m in 2100 according to different SSPs (Table S5). Under SSP5‐8.5 in 2100, the maximum elevation of suitable habitats reaches up to 1844 m (from 1194 m in current conditions). We also found that, under future climate scenarios, the mean elevation of gained habitats is substantially higher than that of lost habitats (Figure 5b, Table S6). The results of two‐way ANOVA and Tukey's HSD test confirmed that both the considered time interval and the climate scenario significantly influence the elevation of suitable habitats (Tables S7 and S8). Because of this elevational shift, most metropolitan areas in Italy are projected to become nearly unsuitable for the survival of this species under future climate scenarios. Although the administrative boundaries of some metropolitan regions, such as Genoa and Turin, include limited mountainous areas that may provide small refugia, lowland urban areas will no longer offer suitable habitats for the species.

## 4 Discussion

**FIGURE 3:**
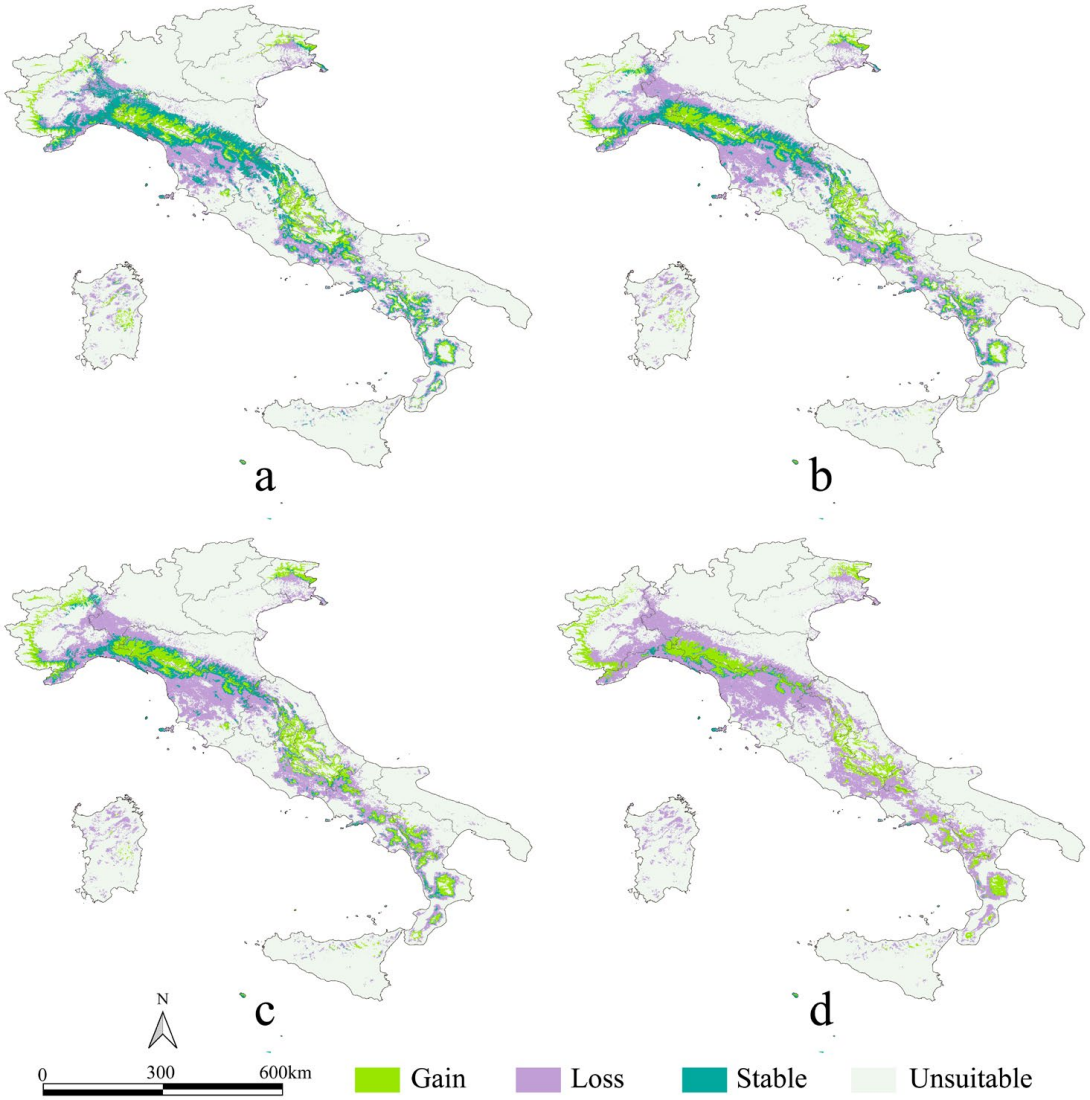
Comparison of future versus current projected habitat suitability for *C. domestica* . (a) SSP2‐4.5 in 2060; (b) SSP5‐8.5 in 2060; (c) SSP2‐4.5 in 2100; and (d) SSP5‐8.5 in 2100. Light green indicates areas that are projected to be unsuitable under current conditions and that will become suitable in the future (indicated as “Gain”); purple indicates areas that are projected to be currently suitable and that will become unsuitable in the future (“Loss”); dark green indicates areas that are projected to be currently suitable and that will remain suitable in the future (“Stable”); gray indicates areas that are projected to be currently unsuitable and that will remain unsuitable in the future (“Unsuitable”).

**FIGURE 4:**
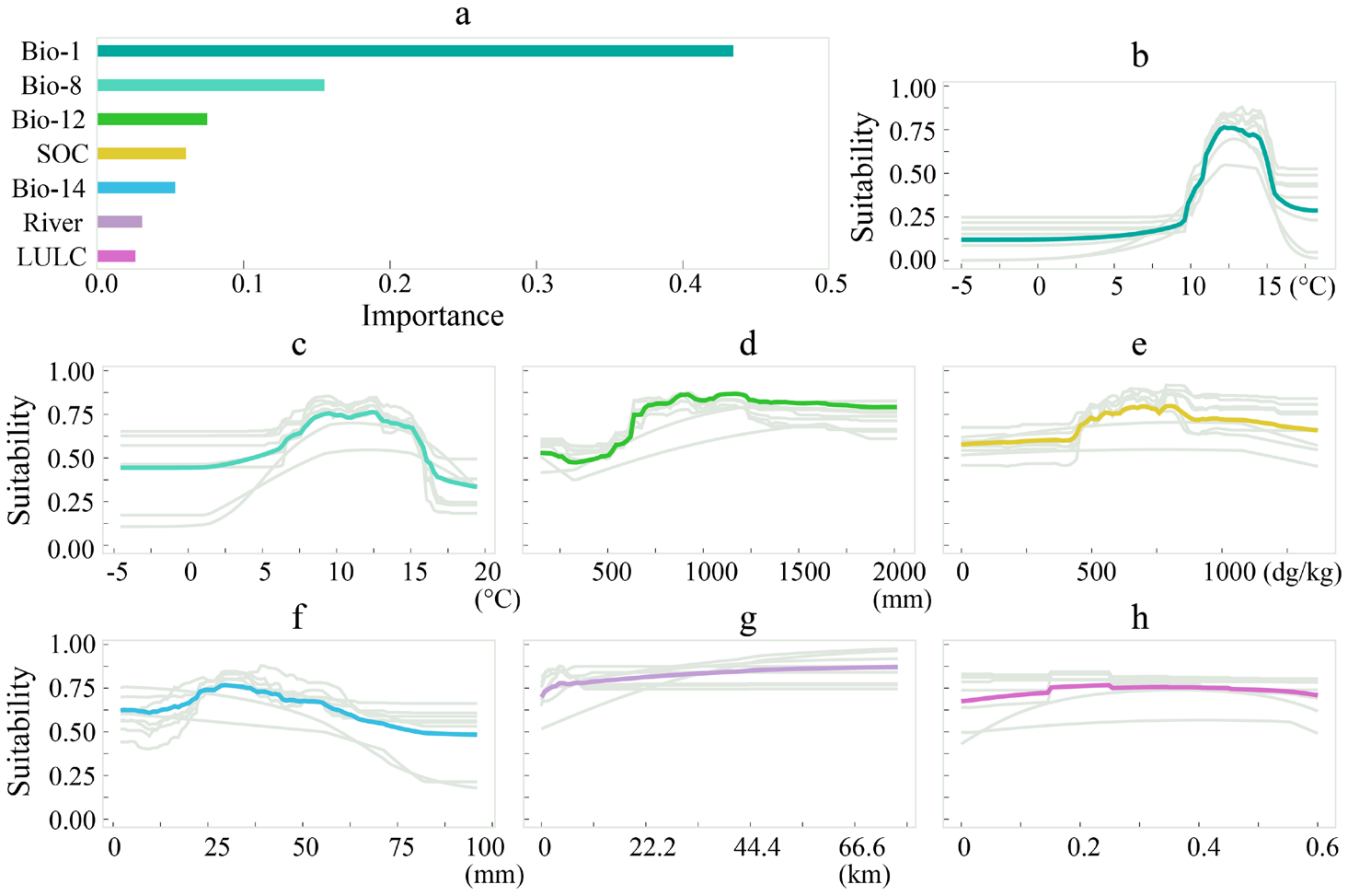
Ranking of environmental variable contributions to the habitat suitability model (a) and response curves (b: Bio‐1, annual mean temperature; c: Bio‐8, mean temperature of wettest quarter; d: Bio‐12, annual precipitation; e: SOC, soil organic carbon; f: Bio‐14, precipitation of the driest month; g: River, distance from the closest river; h: LULC, land use and land cover).

**FIGURE 5:**
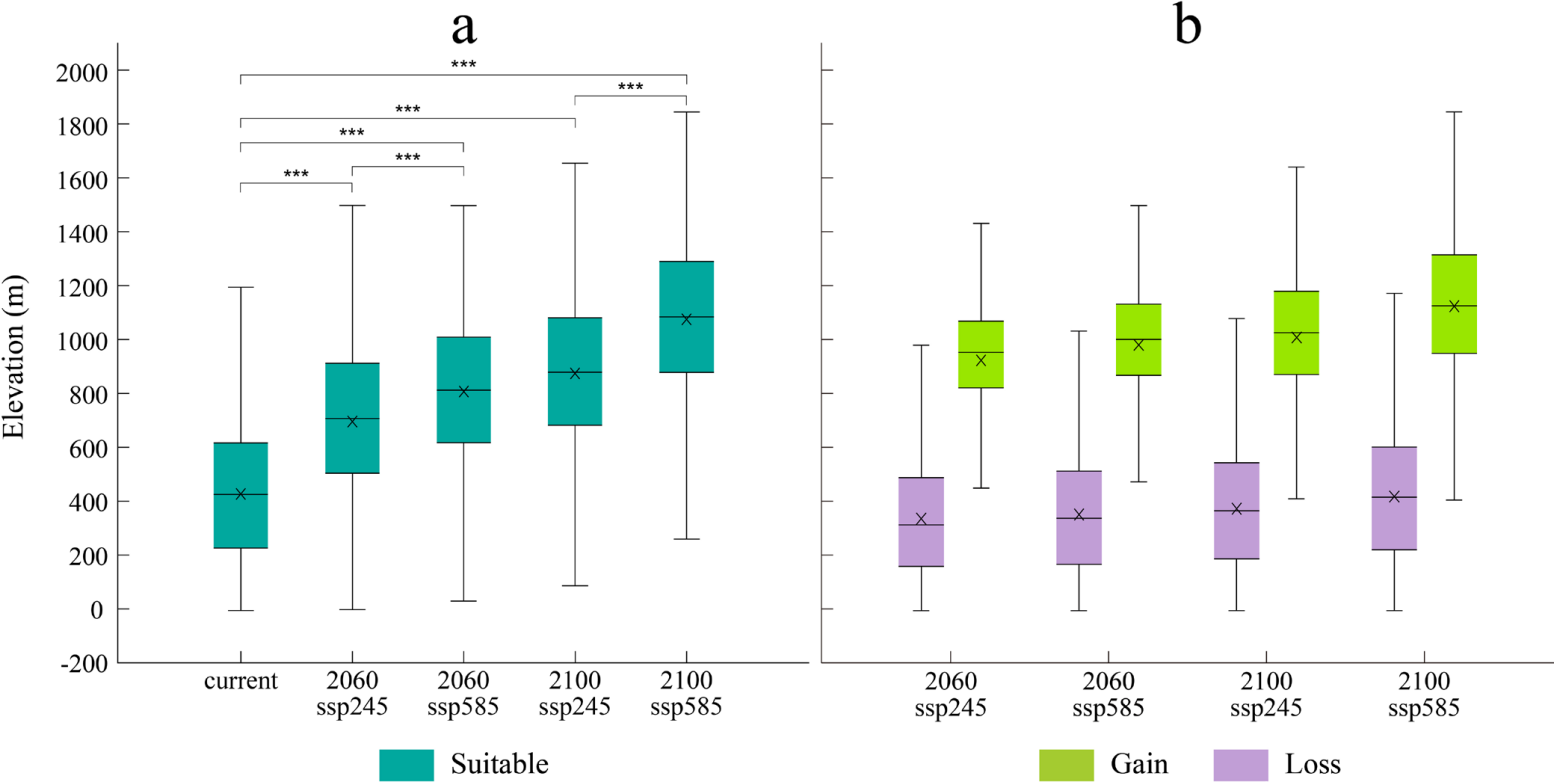
Elevation of *C. domestica* habitats. (a) Elevation of current and future suitable areas; (b) elevation of new and lost habitats. The boxplots represent the 25%–75% range of the elevation data for projected habitats under current conditions and future climate scenarios, with the horizontal line in the center showing the median and the cross marking the mean value. The top and bottom horizontal lines are the maximum and minimum values, respectively. In panel (a), three asterisks indicate strong statistical evidence (*p* < 0.001) of different between‐group means, as obtained from Tukey's HSD test.

In Italy, *C. domestica* is primarily distributed in the mid‐elevation areas of central regions. The species plays an important role in both forest environments and urban areas and is widely considered a highly promising tree species in the context of global climate change, specifically thanks to its thermophilic nature (Paganová 2007) and drought tolerance (Kunz et al. 2018). However, the impact of climate change on the habitat distribution of *C. domestica* is still uncertain. Our habitat projections indicate that, in Italy, the most suitable areas for *C. domestica* are currently located in the Apennine Mountains. Under future climate change, *C. domestica* habitats are projected to suffer severe losses, with the remaining suitable habitats (30% of suitable habitats in the current climate scenario) under SSP5‐8.5 in 2100 still concentrated in the Apennines (Figures 2 and 3), albeit at higher elevations (Figure 5). This upward shift of habitat suitability is projected to become more pronounced as time goes by (i.e., in 2100 vs. 2060) and under more extreme climate scenarios (i.e., with SSP5‐8.5 vs. SSP2‐4.5). The average elevation of suitable habitat will reach 807 and 1075 m under SSP5‐8.5 in 2060 and 2100, respectively. Our study also identifies annual mean temperature as the most significant factor influencing the distribution of this species (Figure 4). Our results can provide scientific support for the conservation and management strategies of this species.

### 4.1 Future Habitat Distribution

Global warming profoundly impacts plant habitat distributions. Our findings indicate that the principal areas of habitat reduction for *C. domestica* in Italy are concentrated in the Apennine Mountains, with significant reductions expected to occur even in core mountainous areas under future climate scenarios. This finding aligns with previous conclusions that climate change represents a primary threat to species within the Rosaceae family (Afifi et al. 2023). The loss of habitat not only threatens the species itself but also raises ecological concerns, including declines in biodiversity, diminished food supply, and reduced carbon storage capacity in ecosystems (Brockerhoff et al. 2017). Interestingly, under future climate scenarios, newly suitable habitats in the northern Apennines tend to converge toward mountain ridges, which indicates an upward migration trend toward higher elevations. Newly gained habitats (e.g., in the Alps in Piemonte region, or in the central and southern Apennines) tend to show a shifting pattern into elevational climatic zonation. Specifically, the variations in temperature and precipitation along elevational gradients create suitable conditions for the ecological requirements of *C. domestica* , enabling the formation of linear, coherent habitats at higher elevations. Although current climatic conditions can sustain suitable habitats for this species in several Italian metropolitan areas, such as Milan, Rome, and Turin, future climate change is projected to lead to the loss of these urban habitats. Under the SSP5‐8.5 scenario, in particular, the species' habitats are predicted to be almost completely lost within major Italian cities, including Milan, Rome, Turin, Venice, Bologna, Cagliari, and Sassari. Although a small number of new habitats may emerge under future climate scenarios, these will be primarily distributed in mountainous climatic zones rather than in urban environments.

### 4.2 Feature Contribution and Functional Response

Although *C. domestica* is known to exhibit some heat and drought tolerance traits (Paganová 2007; Kunz et al. 2018), our results reveal that the annual mean temperature is still the most influential factor in predicting habitat suitability (Figure 4). The species is projected to have a probability of occurrence greater than 0.5 within an annual mean temperature range of approximately 11°C–15°C, aligning with earlier results (P. A. Thomas 2017). Our study indicates that past an annual mean temperature of 12°C, the habitat suitability for the species declines markedly, suggesting that 12°C represents the margin of the optimal temperature range of this species (Paganová 2007). *C. domestica* demonstrates relatively low resistance to extreme drought and high‐temperature events, which can significantly impact its growth rate (Kunz et al. 2018). The threats posed by extreme drought and temperatures to *C. domestica* are manifested in several ways: when temperatures exceed its upper tolerance limit, physiological disruptions occur, including reduced enzyme activity, stomatal closure leading to diminished photosynthetic efficiency, and dehydration due to water stress (Kunz et al. 2016). Although the species can recover radial growth after extreme climatic events, the pressure imposed by climate change on the species is expected to be sustained and long‐lasting. Our results also reveal that the species' habitat suitability increases substantially only when the mean annual precipitation (Bio‐12) exceeds about 500 mm. This is consistent with earlier analyses of mean annual precipitation data from sampling sites of this species (Paganová 2007). The mean temperature of the wettest quarter (Bio‐8) reveals that *C. domestica* can achieve a survival probability above 0.5 only when the minimum temperature remains above 4°C. In addition to the risk of frost caused by low temperatures, reduced evaporation under low‐temperature conditions keeps the soil cold and waterlogged, leading to root hypoxia and inhibiting growth (Jørgensen et al. 2020). The LULC response curve, instead, did not reveal a clear land‐use preference for this species (Figure 4h), which indicates that it can adapt to both urban and forest environments (Schmucker et al. 2024); therefore, we did not introduce LULC projections to evaluate future habitat distributions. Despite the expectedly limited impact of LULC change, its exclusion from future projections may constitute a limitation in our ability to fully account for the future spatial dynamics of *C. domestica* .

### 4.3 Future Habitat Shift to Higher Elevation

Through statistical analysis of habitat distribution under various climate scenarios, we observed a clear trend of *C. domestica* suitable habitat shifting toward higher elevation in response to climate change, with newly formed habitats always at higher altitudes than lost habitats (Figure 5), which is consistent with the general pattern of global mountain species tracking climate change (Lenoir et al. 2008; C. D. Thomas 2010). This trend is expected to be particularly pronounced under the SSP5‐8.5 scenario by the end of the century, with the average elevation of *C. domestica* habitats rising roughly by 648 m compared to current conditions and by 201 m compared to SSP2‐4.5. By 2100, under the SSP5‐8.5 scenario, the species' lowest habitat elevation is projected to rise from sea level to about 260 m, leaving lower elevation areas incapable of supporting its persistence, potentially leading to increased habitat fragmentation or even local extinction. On the other end, we identified 1844 m as the upper elevation limit for the species' survival under SSP5‐8.5 in 2100. Beyond this elevation, factors such as lower temperatures, reduced precipitation, and altered soil composition are projected to hinder its survival.

Climate change will thus render the environmental conditions at lower elevations unsuitable for *C. domestica* , likely forcing the species to migrate to higher elevations to track the spatial shift of suitable habitats. This shift in spatial range would not be without downsides, though. In the Apennines, the stronger winds at higher elevations and the increased risk of organic carbon loss due to low clay content and steep slopes may prove unfavorable for the growth of *C. domestica* seedlings (Tomaselli et al. 2019; Vittori Antisari et al. 2022). A shift in the distribution of *C. domestica* toward higher elevation may also trigger a range of ecological consequences. First, its arrival could alter the composition of existing plant, animal, and microbial communities, possibly disrupting local ecological balances. The 1000–1800 m elevational areas in the Apennine Mountains are primarily dominated by *Fagus sylvatica* (Bonanomi et al. 2020; Buonincontri et al. 2023). *F. sylvatica* typically forms a dense canopy that becomes strongly closed during the growing season, resulting in limited light availability in the understory (Stiers et al. 2019). As a light‐demanding species, *C. domestica* may therefore experience spatial competition with *F. sylvatica* for light resources and canopy space. Moreover, as a newly established species, it may introduce pathogens such as *Phytophthora* that pose threats to native species with slower migration rates (Mullett et al. 2023; Alexander et al. 2015; Pauchard et al. 2016). The involvement of pathogens may lead to pathogen‐mediated competition between *C. domestica* and *F. sylvatica* (Cobb et al. 2010). As a new competitor, *C. domestica* may compete with existing species for space and resources, potentially reshaping the structure and functions of local ecosystems (Alexander et al. 2015).

### 4.4 Conservation Implication

By 2100, *C. domestica* is projected to lose 32%–68% of its habitat throughout Italy, suggesting the need for conservation planning in the long term. Our findings indicate that although the species may gain some high‐elevation climate refuge in the Apennines and the Alps, these gains cannot compensate for the extensive loss occurring in current core areas, particularly in Toscana, where over 9000 km^2^ of habitat may be lost by the end of the century. A sharp upward shift in suitable habitat elevation, which is projected to reach 648 m under the most extreme of the considered climate scenarios, poses a serious challenge to *C. domestica*, given that temperate tree species generally exhibit natural dispersal rates not exceeding 40 km per century (Chan et al. 2024), a rate far slower than required to track such rapid elevational changes in the study area. Therefore, conservation managers should prioritize protecting existing trees in the central Apennines, especially in areas with a mean annual temperature of around 12°C, as these zones may serve as crucial population sources. The emergence of newly suitable habitats suggests that experimental populations could be established at altitudes between 1000 and 1800 m as long‐term refuges to anticipate the occurrence of climate warming. As suitable habitats below 260 m may disappear completely before 2100 under extreme climate scenarios, genetic sampling of lowland populations should be conducted to preserve potentially valuable adaptive traits. Our study predicts habitat loss at a macroecological scale. However, local microclimatic characteristics may buffer the impacts of future climate change, as observed in areas such as Euganean Hills in Veneto and Serra del Prete in the southern Apennines (Gubler et al. 2018; Rita et al. 2021). As a forest fruit species that supports wildlife and provides high‐quality timber, the importance of *C. domestica* extends beyond biodiversity targets alone. This observation suggests that active management interventions, such as assisted migration along elevation gradients, may become a viable component of conservation efforts for this species. Consequently, forest management plans should start to integrate climate adaptation strategies for *C. domestica* rather than wait until habitat loss becomes irreversible, especially in the regions that are projected to be affected the most.

## Author Contributions

**Qianjiang Li:** conceptualization (equal), formal analysis (equal), methodology (equal), visualization (equal), writing – original draft (equal), writing – review and editing (equal). **Zhuoling Li:** formal analysis (equal), writing – original draft (equal), writing – review and editing (equal). **Haozheng Li:** writing – review and editing (equal). **Bohao He:** conceptualization (equal), methodology (equal), project administration (equal), visualization (equal), writing – original draft (equal), writing – review and editing (equal). **Lorenzo Mari:** supervision (equal), writing – original draft (equal), writing – review and editing (equal).

## Funding

This work was supported by the China Scholarships Council (202307560013).

## Conflicts of Interest

The authors declare no conflicts of interest.

## Supporting information


**Figure S1:** Study scope and species occurrence records.
**Figure S2:** Spatial correlation matrix of eight environmental variables.
**Figure S3:** Presence and pseudo‐absence locations of *Cormus domestica* L. in Italy.
**Figure S4:** National and regional administrative boundaries of Italy.
**Table S1:** Performance metrics (mean ± standard deviation) of the test set for the 11 single models and the ensemble model.
**Table S2:** Area occupied by *Cormus domestica* L. habitat within each administrative region of Italy under current climate conditions.
**Table S3:** Projected habitat area of *Cormus domestica* L. under current conditions and changes under future climate scenarios.
**Table S4:** Projected loss of *Cormus domestica* L. habitat area within each administrative region of Italy under future climate scenarios.
**Table S5:** Maximum, minimum, mean, and median elevations of *Cormus domestica* L. habitat under current conditions and future climate scenarios.
**Table S6:** Mean elevations of gained and lost habitat under future climate scenarios.
**Table S7:** Two‐way ANOVA results for the effects of Year and SSP on habitat elevation.
**Table S8:** Results of Tukey HSD test comparing suitable habitat elevations under current and projected future climate scenarios.

## Data Availability

The data supporting this study are openly available in Figshare at https://figshare.com/s/b1119f044bc2b4bddbad.
