# Supplementary material for: Climate Change Alters Elevational Distribution Patterns of Cormus domestica Habitat
Source: Ecol Evol. 2026 Apr 29;16(5):e73602. doi: 10.1002/ece3.73602 (PMC13125968; doi:10.1002/ece3.73602)
Supplement: Supplementary file 1 — Figure S1: Study scope and species occurrence records. Figure S2: Spatial correlation matrix of eight environmental variables. Figure S3: Presence and pseudo‐absence locations of Cormus domestica L. in Italy. Figure S4: National and regional administrative boundaries of Italy. Table S1: Performance metrics (mean ± standard deviation) of the test set for the 11 single models and the ensemble model. Table S2: Area occupied by Cormus domestica L. habitat within each administrative region of Italy under current climate conditions. Table S3: Projected habitat area of Cormus domestica L. under current conditions and changes under future climate scenarios. Table S4: Projected loss of Cormus domestica L. habitat area within each administrative region of Italy under future climate scenarios. Table S5: Maximum, minimum, mean, and median elevations of Cormus domestica L. habitat under current conditions and future climate scenarios. Table S6: Mean elevations of gained and lost habitat under future climate scenarios. Table S7: Two‐way ANOVA results for the effects of Year and SSP on habitat elevation. Table S8: Results of Tukey HSD test comparing suitable habitat elevations under current and projected future climate scenarios. [file ECE3-16-e73602-s001.docx]

**Climate change drives elevational gradients in**

***Cormus domestica* (L.) habitat**

Supplementary Information:

Figs. S1-S4, Tables S1-S8


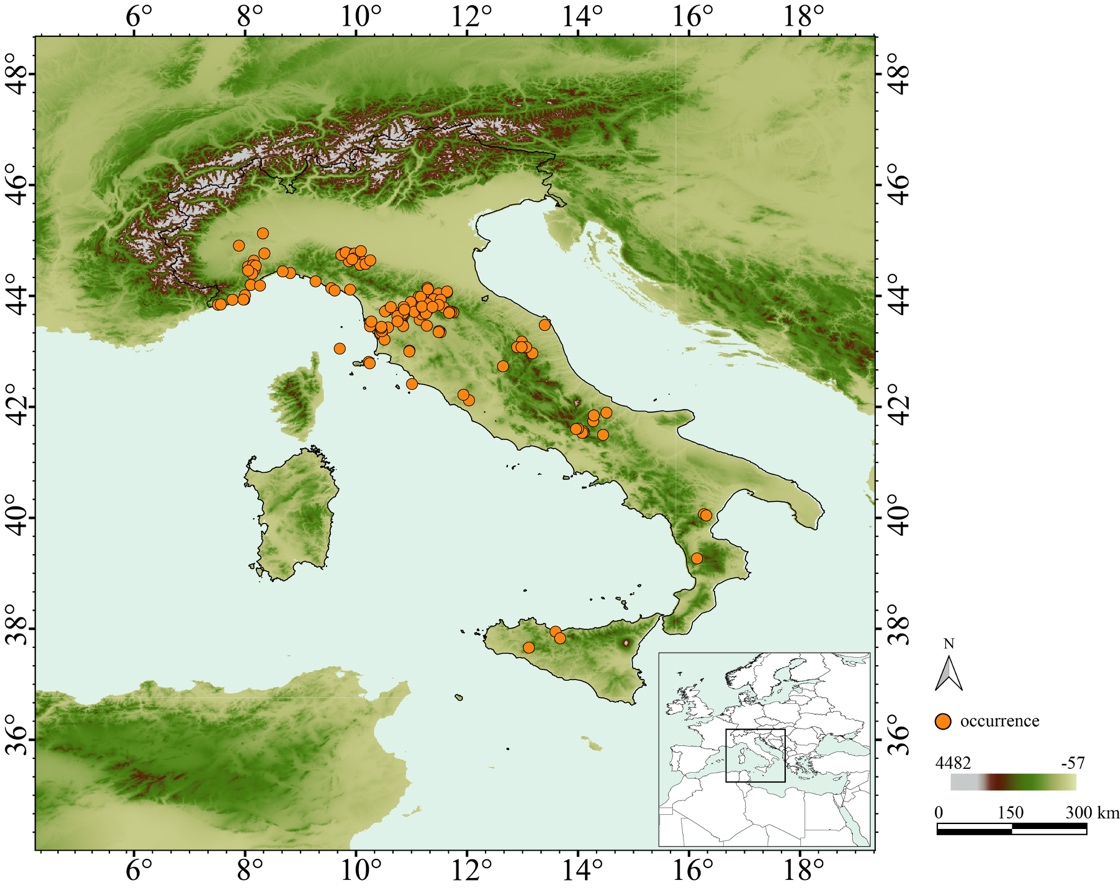


Fig. S1. Study scope and species occurrence records.


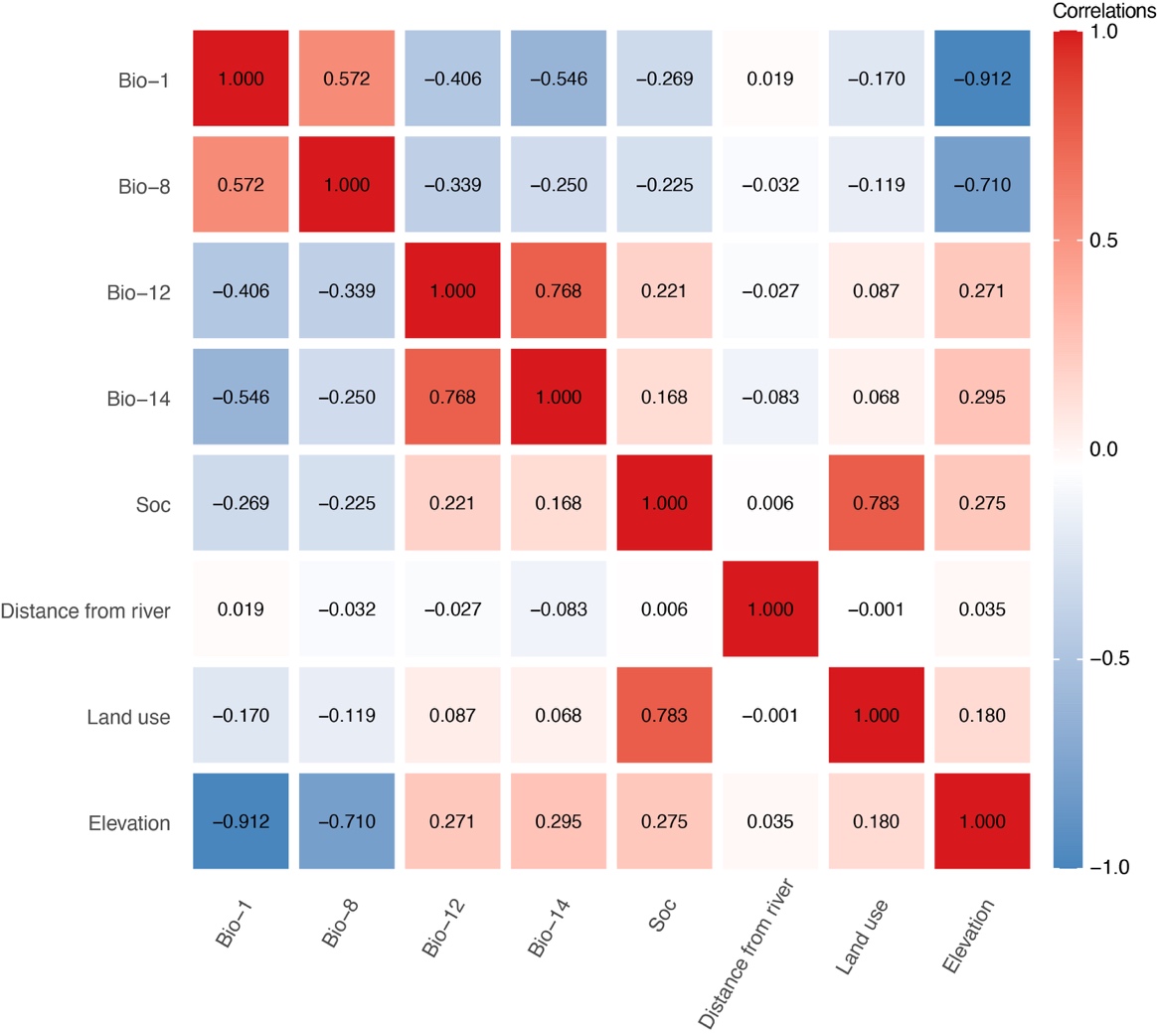


Fig. S2. Spatial correlation matrix of 8 environmental variables.


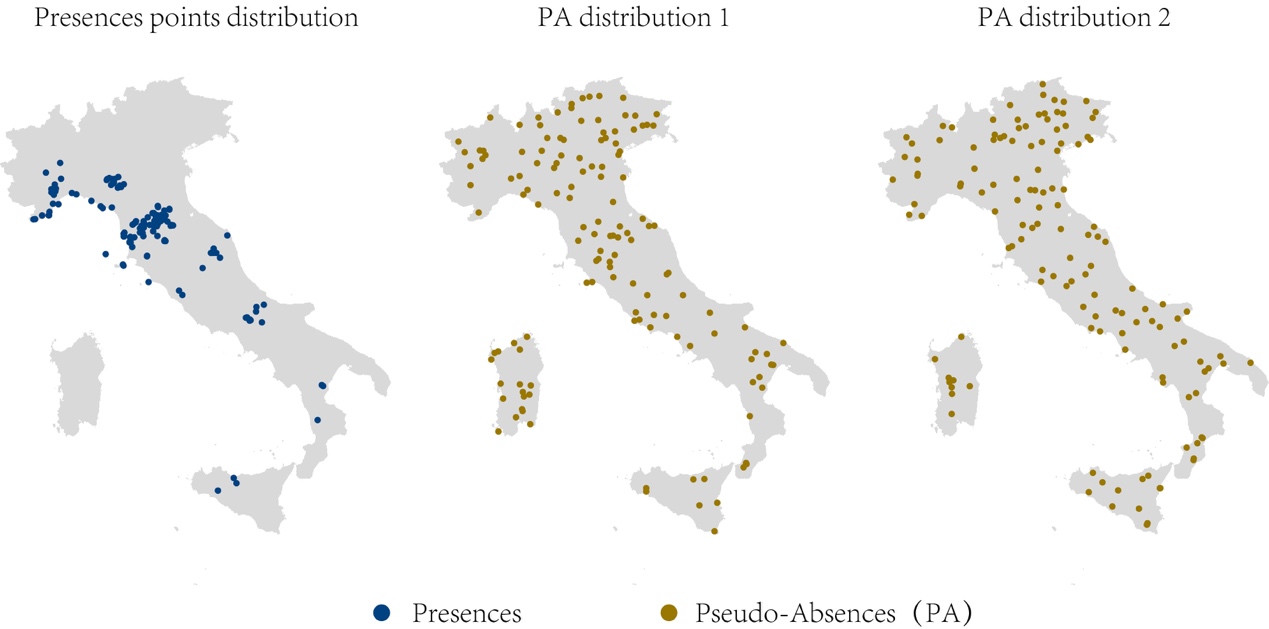


Fig. S3. Presence and pseudo-absence locations of *Cormus domestica* L. in Italy. The blue points in panel (a) indicate locations where the species has been observed, while yellow points in panels (b-c) represent two realizations of pseudo-absence locations (1:1 match to the number of retained observation records).


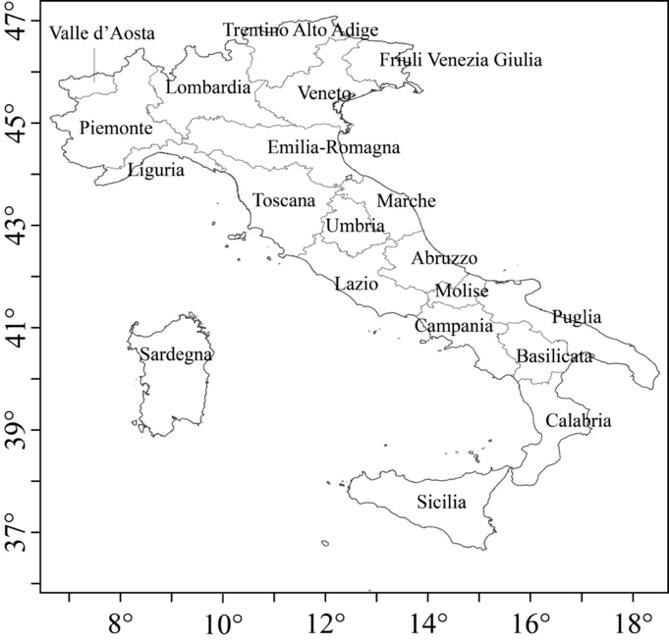


Fig. S4. National and regional administrative boundaries of Italy.

Table S1. Performance metrics (mean±standard deviaiton) of the test set for the 11 single models and the ensemble model.

| Models | TSS | AUC |
| --- | --- | --- |
| ANN | 0.41±0.17 | 0.72±0.09 |
| CTA | 0.41±0.10 | 0.73±0.07 |
| FDA | 0.42±0.12 | 0.78±0.06 |
| GAM | 0.26±0.09 | 0.71±0.06 |
| GBM | 0.58±0.10 | 0.85±0.06 |
| GLM | 0.49±0.15 | 0.82±0.07 |
| MARS | 0.43±0.12 | 0.78±0.07 |
| MAXNET | 0.55±0.11 | 0.84±0.06 |
| RF | 0.59±0.11 | 0.86±0.06 |
| SRE | 0.42±0.13 | 0.71±0.06 |
| xGBOOST | 0.45±0.09 | 0.80±0.06 |
| Ensemble | 0.75±0.05 | 0.91±0.03 |

Table S2. Area occupied by *Cormus domestica* L. habitat within each administrative region of Italy under current climate conditions.

| District | Area (km^2^) |
| --- | --- |
| Toscana | 14,712.91 |
| Emilia-Romagna | 6,621.64 |
| Piemonte | 6,526.35 |
| Campania | 5,441.08 |
| Lazio | 5,043.93 |
| Lombardia | 4,301.44 |
| Liguria | 4,213.19 |
| Calabria | 4,075.69 |
| Umbria | 3,232.16 |
| Sardegna | 2,744.84 |
| Basilicata | 2,402.05 |
| Marche | 2,103.4 |
| Friuli-Venezia Giulia | 1,848.87 |
| Abruzzo | 1,812.41 |
| Molise | 1,292.48 |
| Sicilia | 1,025.8 |
| Veneto | 579.41 |
| Puglia | 209.76 |

Table S3. Projected habitat area of *Cormus domestica* L. under current conditions and changes under future climate scenarios.

| Scenarios | Years | Area (km^2^) | Area changes (km^2^) |
| --- | --- | --- | --- |
| Current | 1970-2020 | 68,206.04 | NA |
| SSP2-4.5 | 2041-2060 | 46,584.60 | -21,621.44 |
| SSP5-8.5 | 2041-2060 | 37,534.94 | -30,671.10 |
| SSP2-4.5 | 2081-2100 | 34,737.59 | -33,468.45 |
| SSP5-8.5 | 2081-2100 | 21,552.99 | -46,653.05 |

Table S4. Projected loss of *Cormus domestica* L. habitat area within each administrative region of Italy under future climate scenarios.

| District | 2041-2060 | | | 2081-2100 | |
| --- | --- | --- | --- | --- | --- |
|  | SSP2-4.5 | SSP5-8.5 | SSP2-4.5 | | SSP2-8.5 |
| Toscana | 9,068.21 | 1,0621.37 | 11,585.38 | | 13,642.62 |
| Piemonte | 3,848.36 | 4,708.74 | 5,109.18 | | 6,371.93 |
| Campania | 3,833.01 | 4,339.00 | 4,698.50 | | 5,363.78 |
| Lazio | 3,378.19 | 3,905.93 | 4,404.25 | | 5,040.74 |
| Sardegna | 2,626.56 | 2,726.35 | 2,745.54 | | 2,745.54 |
| Calabria | 2,555.55 | 2,996.30 | 3,291.83 | | 3,982.69 |
| Lombardia | 2,332.94 | 3,857.32 | 3,976.30 | | 4,285.27 |
| Emilia-Romagna | 1,697.73 | 3,450.47 | 4,388.26 | | 6,296.44 |
| Liguria | 1,626.09 | 2,185.81 | 2,571.54 | | 3,700.59 |
| Friuli-Venezia Giulia | 1,519.90 | 1,587.06 | 1,672.14 | | 1,849.34 |
| Basilicata | 1,425.86 | 1,775.77 | 2,036.76 | | 2,401.39 |
| Umbria | 1,349.10 | 2,255.54 | 2,722.51 | | 3,218.27 |
| Abruzzo | 1,201.97 | 1,387.48 | 1,557.00 | | 1,812.87 |
| Sicilia | 825.84 | 873.17 | 900.04 | | 940.34 |
| Marche | 782.98 | 1,313.92 | 1,588.34 | | 2,090.50 |
| Molise | 719.65 | 918.59 | 1,031.82 | | 1,292.81 |
| Veneto | 561.65 | 576.36 | 578.28 | | 579.56 |
| Puglia | 209.82 | 209.82 | 209.82 | | 209.82 |

Table S5. Maximum, minimum, mean, and median elevations of *Cormus domestica* L. habitat under current conditions and future climate scenarios.

| Scenarios | Years | Maximum (m) | Minimum  (m) | Mean  (m) | Median  (m) |
| --- | --- | --- | --- | --- | --- |
| Current | 1970-2020 | 1,194 | -6 | 427 | 425 |
| SSP2-4.5 | 2041-2060 | 1,498 | -2 | 695 | 707 |
| SSP5-8.5 | 2041-2060 | 1,497 | 29 | 807 | 812 |
| SSP2-4.5 | 2081-2100 | 1,654 | 86 | 874 | 879 |
| SSP5-8.5 | 2081-2100 | 1,844 | 260 | 1075 | 1084 |

Table S6. Mean elevations of gained and lost habitat under future climate scenarios.

| Scenarios | Years | Gain (m) | Loss (m) |
| --- | --- | --- | --- |
| SSP2-4.5 | 2041-2060 | 923 | 336 |
| SSP5-8.5 | 2041-2060 | 980 | 351 |
| SSP2-4.5 | 2081-2100 | 1008 | 372 |
| SSP5-8.5 | 2081-2100 | 1124 | 418 |

Table S7. Two-way ANOVA results for the effects of Year and SSP on habitat elevation.

|  | Df | Sum Sq | Mean Sq | F value | Pr (>F) |
| --- | --- | --- | --- | --- | --- |
| Year | 2 | 1.38E+10 | 6.89E+09 | 94197 | <2e-16 |
| SSP | 2 | 1.24E+09 | 6.21E+08 | 8501 | <2e-16 |
| Residuals | 326117 | 2.38E+10 | 7.31E+04 |  |  |

Table S8. Results of Tukey HSD test comparing suitable habitat elevations under current and projected future climate scenarios.

|  | Difference | Lower | Upper | p adj |
| --- | --- | --- | --- | --- |
| 2100 ssp585 - 2100 ssp245 | 200.68 | 195.57 | 205.80 | < 0.001 |
| 2060 ssp245 - 2100 ssp245 | -178.75 | -182.94 | -174.57 | < 0.001 |
| 2060 ssp585 - 2100 ssp245 | -67.06 | -71.45 | -62.67 | < 0.001 |
| Current - 2100 ssp245 | -447.41 | -451.30 | -443.52 | < 0.001 |
| 2060 ssp245 - 2100 ssp585 | -379.44 | -384.30 | -374.58 | < 0.001 |
| 2060 ssp585 - 2100 ssp585 | -267.74 | -272.78 | -262.70 | < 0.001 |
| Current - 2100 ssp585 | -648.09 | -652.70 | -643.48 | < 0.001 |
| 2060 ssp585 - 2060 ssp245 | 111.70 | 107.60 | 115.79 | < 0.001 |
| Current - 2060 ssp245 | -268.66 | -272.20 | -265.11 | < 0.001 |
